# Post-surgery fluids promote transition of cancer stem cell-to-endothelial and AKT/mTOR activity, contributing to relapse of giant cell tumors of bone

**DOI:** 10.18632/oncotarget.18783

**Published:** 2017-06-28

**Authors:** Flavio Fazioli, Gianluca Colella, Roberta Miceli, Mariano Giuseppe Di Salvatore, Michele Gallo, Serena Boccella, Annarosaria De Chiara, Carlo Ruosi, Filomena de Nigris

**Affiliations:** ^1^ Division of Musculoskeletal Oncology Surgery, National Cancer Institute G. Pascale, Naples, Italy; ^2^ Department of Human Health, Federico II University of Naples, Naples, Italy; ^3^ S.C. Cellular Biology and Biotherapy, National Cancer Institute G. Pascale, Naples, Italy; ^4^ Department of Experimental Medicine, University of Campania “Luigi Vanvitelli”, Naples Italy; ^5^ Division of Pathology, National Cancer Institute G. Pascale Foundation, Naples, Italy; ^6^ Department of Biochemistry, Biophysics and General Pathology, University of Campania “Luigi Vanvitelli”, Naples Italy

**Keywords:** recurrences, sarcoma, angiogenesis, stem cells, AKT

## Abstract

Giant cell tumors of bone (GCTB) are rare sarcomas with a high rate of unpredictable local relapse. Studies suggest that surgical methods affect recurrence, supporting the idea that local disease develops from re-growth of residual cancer cells. To identify early prognostic markers of individual risk of recurrence, we evaluated the effect of post-surgery fluids from a cohort of GCTB patients on growth of primary and established sarcoma cell lines, and mice xenograph. Post-surgery fluids increased cell growth and enhanced expression of CD44^++^, the principal receptor for the extracellular matrix component hyaluronan and the mesenchymal stem marker CD117^+^. Cancer cells became highly invasive and tumorigenic, acquiring stemness properties, and activated AKT/mTOR pathway. Prolonged stimulation with post-surgery fluids down-regulated the mesenchymal gene *TWIST1* and Vimentin protein, and transdifferentiated cells into tubule-like structures positive to the endothelial markers VE-Cadherin and CD31^+^. In mice, post-surgery fluids gave rise to larger and more vascularized tumors than control, while in patients AKT/mTOR pathway activation was associated with recurrence by logistic regression (Kaplan-Meier; P<0.001). These findings indicate that post-surgery fluids are an adjuvant in mechanisms of tumor regrowth, increasing stem cell growth and AKT/mTOR activity.

## INTRODUCTION

Giant cell tumor of bone (GCTB) is a rare, locally aggressive and vascularized tumor [1]. The hallmarks of this tumor are its aggressively lytic behavior, and high potential to local relapse (20%–50% of cases), correlated to type of surgical treatment and local presentation of the tumor [2, 3]. GCTB comprises three different cellular components: spindle-like stromal cells, some rounded mononuclear cells and osteoclast-like multinucleated giant cells [4]. Stromal cells perform an essential function in the recruitment of tumor-associated myeloid lineage cells and formation of osteoclast-like giant cells responsible for bone desorption [5, 6]. Several features of stromal cells suggest their neoplastic role in GCTB. Most notably, these cells are highly proliferative, allowing *in vitro* propagation through numerous passages in monolayer cell culture [4, 7, 8], and have demonstrated a capacity to form tumors when implanted in immune-compromised mice [5].

GCTB has been classified into three grades by its histological appearance [9]. However, the clinical and prognostic value of tumor grading has been disputed [10, 11]. Apparently benign lesions after surgery can therefore develop unpredictable recurrences [9]. Most relapses occur at or close to the same site of the primary cancer. The hypothesis that local disease may develop from regrowth of residual cancer cells [6] is supported by the observation that a small subpopulation (1%) of GCTB cells have a stem-like phenotype [12]. Other clinical and experimental data seem to confirm the concept of tumor dormancy of malignant lesions due to similarities between the stroma at sites of wound repair and reactive stroma in cancer [7]. However, the mechanism by which GCTB cells are restrained from establishing dormancy is poorly investigated. Prognosis of GCTB and evaluation of individual risk of recurrence is therefore a hot research topic. Surgical and wide resection of the tumor is often the preferred treatment, although sometimes impractical [12], and therapeutic options are continuously being explored, including denosumab [13] and bisphosponates [14]. Even with this multidisciplinary approach, treatment results are still unsatisfying, and the behavior of GCTB at first diagnosis remains unpredictable in term of prognosis. As several studies reported that surgical methods affect recurrence rate [3], we postulated that post-surgery fluids, consisting in acute wound fluids) may contribute to regrowth of residual tumor cells and neoangiogenesis. Our previous studies demonstrated that sarcoma vascularization plays a key role in tumor growth and metastasis [15, 16] and defined, in some instances, the mechanism [17–19].

In this study we explored the role of post-surgery wound fluids (WFs) from GCTB patients as an adjuvant effector of recurrence in the mechanism of growth, invasiveness and neoangiogenesis in primary GCTB, established cell lines, and mice model. In order to define potential targets we also investigated the molecular pathway involved in transdifferentiation of mesenchymal/cancer stem cells into endothelial-like structures, which supports tumor vascularization *in vivo*.

## RESULTS

### Histopathology results

**Figure 1:**
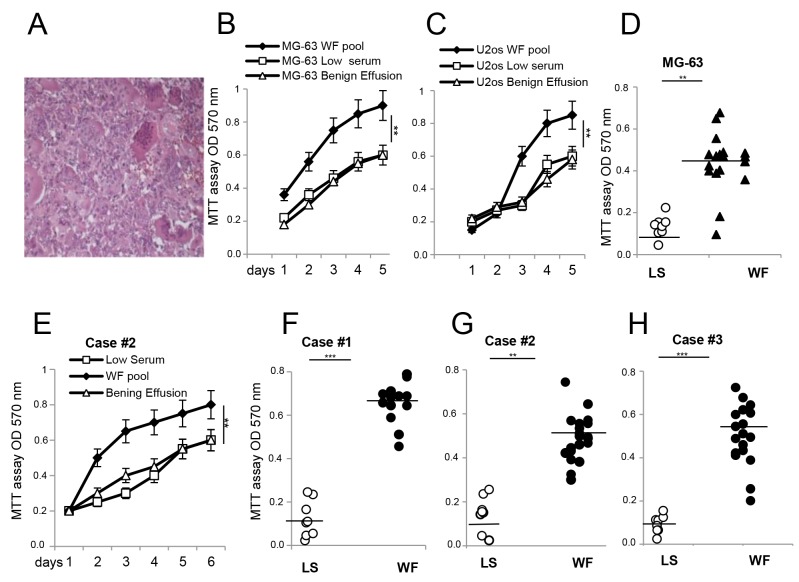
Proliferative effects of malignant WFs on established cell lines and primary cultures from GCTB patients **(A)** Representative H&E of GCTB specimen at 40x magnification. **(B)** MG-63 cells. **(C)** U2os cells grown in presence of WF pool (black square), LS (white square) and benign effusion (white triangle) dosed by MTT assay at different time points and reading absorbance at 570 nm. **(D)** MTT assay of MG-63 cell line grown for 48h with WF from single patient (n=20, black triangle) or LS (n=9, white circle). **(E)** GCTB cell line case#2 grown in presence of WF pool (black square), LS (white square) and benign effusion (white triangle) dosed by MTT assay followed for several days as indicated. **(F)** Primary GCTB cell line case#1. **(G)** GCTB cell line case#2. **(H)** GCTB cell line case#3, all grown for 48h with WFs from single patient (n=20, black circle) or LS (n=9, white circle). Each dot represents one WF tested. All the experiments were performed in triplicate, and statistical analyses were performed using the Student’s t-test: **P <0.01; ***P <0.001 vs LS.

In order to characterize whether post-surgery wound fluids interact with tumor cells, patients affected by GCTB (n=56) were enrolled in the study (Table 1). All patients had resectable disease and naïve for any therapeutic treatments or others surgery. Specimens were evaluated for tumor morphology and disease characteristics. Baseline tumor samples were typically composed of tumor stromal giant cells, and sparse focal areas made up of bone matrix and fibro-osseous tissue (Figure 1A). The mean age of patients was 48 years. GCTB were preferentially localized in inferior arms and 32% of patients developed relapse in the same area of the first tumor with median time of 16.2 months (Table 1). The frequency and time of relapse was not statistically related to tumor site, surgical treatment or tumor grade. A subset of post-surgery wound fluids (WFs) from primary tumor patients (n=20) was randomly selected and pooled to assess the effect on sarcoma cells. The biochemical characteristics of WFs are summarized in Supplementary Table 1.

**Table 1:** Baseline patient characteristics at time of resection and IORT (*n* = 56)

Characteristics	Number of patients	Total percentage
**Age**	Median (48 years)	
	Range (19-67 years)	
**Gender**	M (n=24)	43
	F (n=32)	57
**Site**		
Inferior arms	(n=47)	83
Superior arms	(n=9)	7
**Histology**		
Giant cell tumor of bone GCT	(n=56)	100
**AJCC group stage**		
I	(n=35)	62
II	(n=21)	38
**Local recurrence type**		
Single	(n=14)	25
Multiple	(n=4)	7
Total percentage of recurrences		**32**
**Time to recurrence**		
At follow up range (12-25mo.)	(n=56)	
Median	**16.2mo**	
**AKT/mTOR above cut/off**	22	39

### Proliferative capabilities of wound fluids are not patient specific

To verify whether WFs affect the proliferation of tumor cells, we tested patient fluids individually and as a pool, and carried out a proliferation assay. The pool of WFs increased proliferation of two sarcoma cell lines (MG-63 cells and U2os cells, Figure 1B and 1C) compared to a pool of benign effusion or low serum (LS), while no significant differences were observed for normal osteoblastic cells (Supplementary Figure 1). Since benign effusion pool and low serum gave same results, we used LS as control in subsequent experiments. To assess if individual WFs could affect cell proliferation differently, the experiments were repeated testing each WF by MTT assay after 48h. Data indicated that MG-63 cells grew faster in the majority of WFs tested (n=20) than LS (n=9), showing that proliferative capability of WFs are not patient-specific (Figure 1D). We then examined the biological interaction between sarcoma WFs and patient-derived cells. Primary cultures of tumor cells are established from GCTB patients and grown as previously reported [20, 21]. GCTB cells stimulated with WF pool at different time point grew faster than in LS (Figure 1E). However, patients responded differently to WFs tested, with a proliferation rate ranging from a median of 2- to 5-fold (over LS controls) after 48h (Figure 1F-1H). In addition, the WF pool induced a consistent and significant increase in invasion capability of cells tested by scratches, transwell assays and TEM images (Supplementary Figure 2).

### Activation of stem cell markers in cells from patients and established cell lines

**Figure 2:**
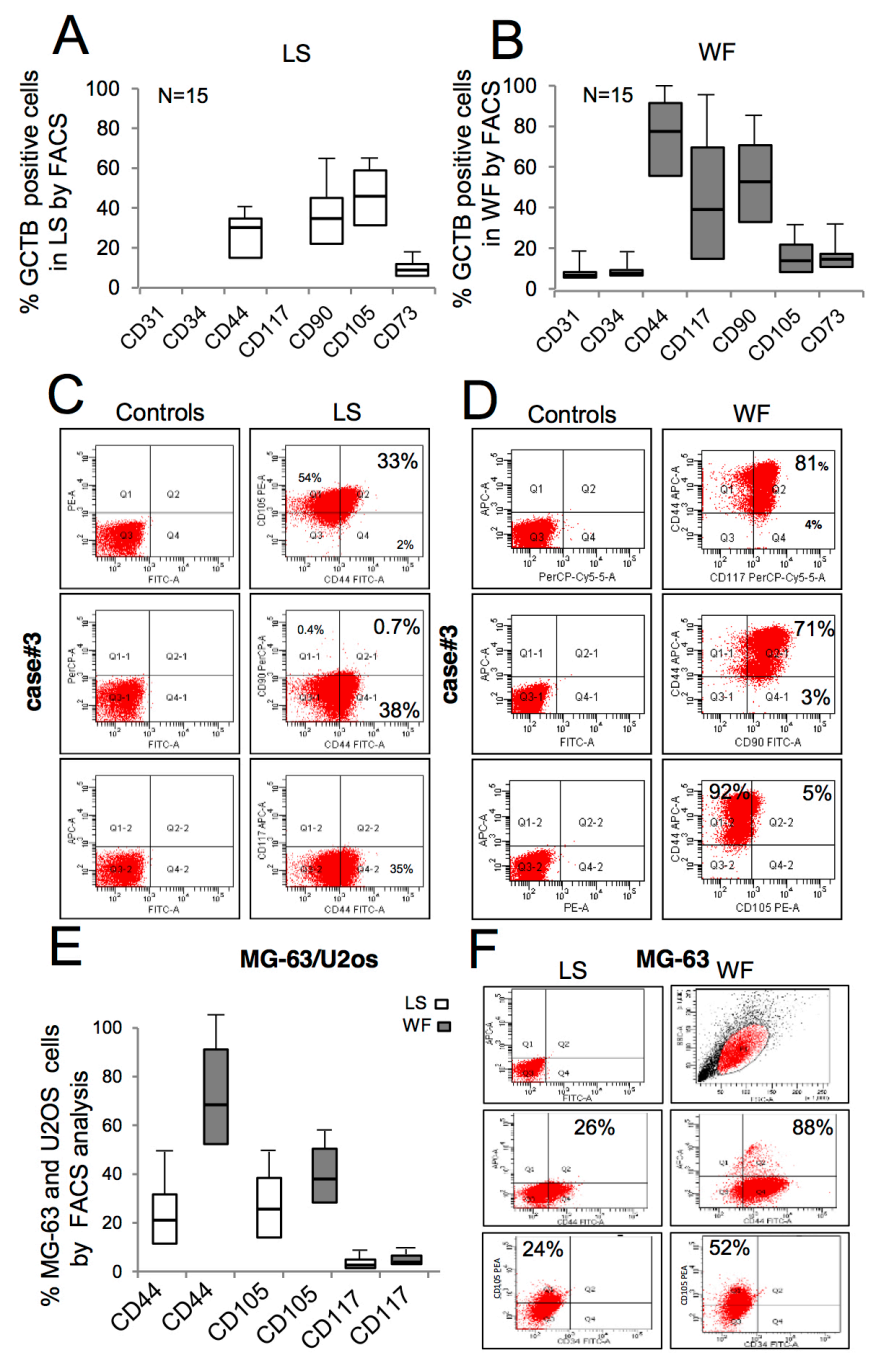
FACS immunophenotypic features of *ex vivo* GCTB cells and *in vitro* expanded sarcoma cells after WF pool stimulation **(A)** Box plots reporting the percentage of GCBT cells positive to mesenchymal markers, as indicated, following stimulation for 48h with LS analyzed by FACS (mean ± SD of 15 independent primary cell lines). **(B)** Percentage of GCBT cells positive to antigens, as indicated, following 48h treatment with WF pool analyzed by FACS (mean ± SD of 15 independent primary cell lines). **(C)** Representative GCTB primary cell line from case#3 grown in LS for 48h labeled with monoclonal anti-CD44, CD105 and CD117 gated by FACS and matched controls. **(D)** GCTB primary cell line from patient (case#3) grown in presence of WF pool for 48h labeled with monoclonal anti-CD44, CD105 and CD117 gated by FACS. **(E)** Percentage of MG-63and U20s cells positive to markers, as indicated, following stimulation for 48h with LS (white box) and WF pool (gray boxes) analyzed by FACS (mean ± SD of 3 independent experiments). **(F)** Right panel, representative FACS plots of MG-63 gated with anti-CD44, CD117 and CD105 antibodies following growth for 48h in presence of LS. Left panel, MG-63 cells stimulated with WF pool for 48h and then analyzed by FACS.

To investigate whether WFs affect the expression of antigens involved in tumor progression, established cells and primary GCTB cells were analyzed by flow cytometry (Figure 2). Since we did not observe any significant different biological effects between sera tested, we used only the pool of WFs in subsequent experiments. The majority of primary GCTB cell lines (n=15) exposed for 48h to LS were positive to MSC markers such as CD105 and CD44, with a median of 42% and 29%, respectively, across a variety of GCTBs. We did not detect CD45, CD34, CD31 and CD117 antigensw in LS, and found no significant differences between different passages in each patient (Figure 2A). A representative immunophenotype of primary GCTB cells grown in LS is shown in Figure 2C. Following WF pool stimulation CD44 antigen increased to a median of 70% in GCTB cells cultured for 48h with WF pool, compared to 30% in LS-treated cells (Figure 2B). More importantly, 29% of cells become positive to the mesenchymal stem marker CD117 in WF-treated cells, whereas this antigen was undetectable in cells treated with LS (Figure 2B). A representative FACS analysis performed on GCTB cells following WF pool stimulation is shown in Figure 2D. In MG-63 and U2os cells, WF pool enhanced expression of CD44 (median of 88% in WF pool versus 26% in LS) and CD105 (52% in WF pool versus 24% in LS) (Figure 2E). A representative FACS analysis is shown in Figure 2F. No differences were observed for other mesenchymal markers (CD90 and CD73) data not shown.

### Wound fluid promotes growth of stem cells

**Figure 3:**
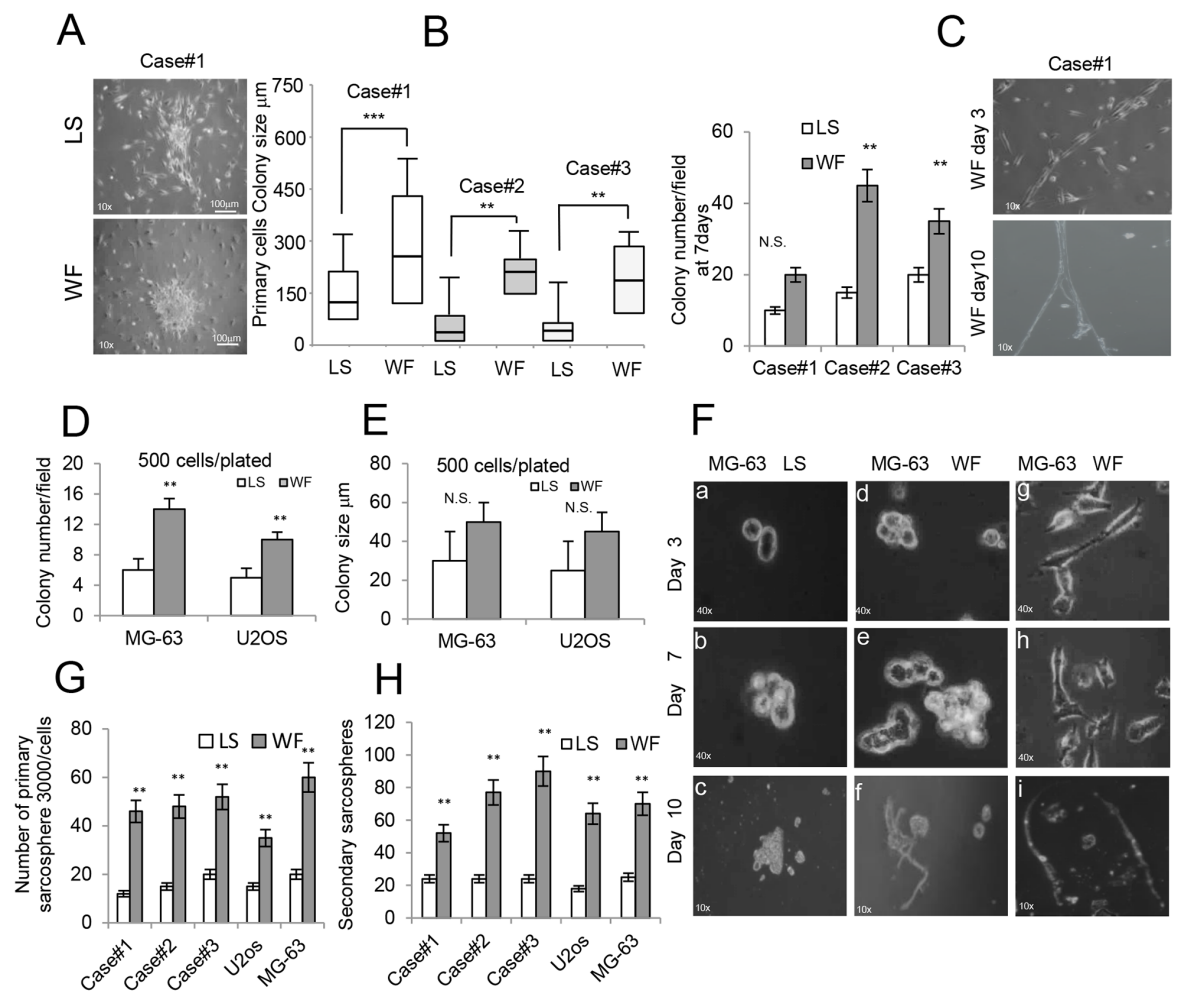
Effect of WF pool colony formation and sarcospheres **(A)** Representative colony clusters formed by primary GCBT cell line stimulated for 48h with LS (upper panel) and with WF pool (lower panel) at 10x magnification. **(B)** Left panel, box plots reporting colony size per field (500 cells plated) of 3 different primary GCTB cell lines in LS and WF pool as indicated after 48h. right panel, colony number per field of 3 different primary GCTB cell lines in LS and WF pool as indicated after 7days. Mean ± SD of 3 independent experiments: **P <0.01; ***P <0.001. **(C)** Image of primary GCTB cell line grown in WF pool showing a subpopulation of cells with a cobblestone morphology at day 3 (upper panel) and after 10 days (lower panel) at 10x magnification. **(D)** Bar graph of MG-63 and U2os colony numbers per field, 500 cells plated, grown for 7 days in presence of LS (white box) or WF pool (gray box). The cells were stained with crystal violet and colonies >20μm were counted. **(E)** Bar graph reporting colony size per field (500 plated cells) of MG-63 and U2os cells grown for 7 days in presence of LS (white box) or WF pool (gray box). **(F)** Sarcosphere assay of MG-63 cells grown in LS (panels a-c) and WF pool at different time points as indicated (panels d-i) at 10x magnification. At day 7 colonies were evident (panels b, e), and in presence of WF pool spontaneously differentiated into a tubular-like phenotype at day 10 (panels f) at 10x magnification. Panels (g-i) a subpopulation of MG-63 cells grown in WF pool showing cobblestone morphology and organized in tubular-like structures at day 10 (panel i). 10x magnification. **(G)** Number of spheres (>20 μm in diameter) generated after 7 days from 3000 plated cells treated with WF pool compared with LS group. **(H)** 3000 single cells isolated from primary spheres were cultured in sphere-forming specific system with WF pool or LS to form secondary spheres. The number of secondary spheres after 7 days (>20 μm in diameter) are reported. All the experiments were performed in triplicated and statistical analyses were performed using the Student’s t-test: ** P <0.01; ***P <0.001.

Since CD117 and CD44 are both associated with cancer mesenchymal stem cells (MSCs) and play a mechanistic role in regulating malignant/metastatic behavior, we tested the capability of WF pool to stimulate tumorigenesis *in vitro*. The oncogenic properties of stimulated cells were compared by colony assay. GCTB primary cells plated at low density (500 cell/well) after 48h culturing in WF pool formed colonies larger (median 300 μm in diameter) than in LS (125 μm in diameter). A representative image is shown in Figure 3A, and size and number quantifications are reported in Figure 3B. Similarly, a subpopulation of cells with cobblestone morphology was also evident when cultured in WF pool (Figure 3C). These cells appeared aligned after three days, and at day 10 developed a tubular-like morphology, as shown in Figure 3C (upper and lower panels). Consistently, MG-63 and U2os cells grown in WF pool formed a higher number of colonies (3-fold and 2-fold, respectively) compared to LS (Figure 3D). In contrast, no significant differences were observed in colony size (Figure 3E). To investigate the effect of WF pool on proliferation and number of stem-like cells, we performed primary and secondary sarcosphere assays and counted the number of spheres (>20μm in diameter). A representative image of MG-63 spheres is shown in Figure 3F. WF pool increased the formation of primary and secondary spheres in all cell lines compared to control (P<0.01) (Figure 3G). Moreover, MG-63 spheres clearly differentiated into tubule-like structures similar to vessels at 10 days following WF pool stimulation (Figure 3F, lower panel f). Additionally, we observed a subpopulation of MG-63 cells with fibroblast-like phenotype assuming a tubule-like phenotype at day 10 (Figure 3F, panels g-i). To confirm the effect of WF pool on growth of stem-like cells, we dosed the expression levels of some markers of cancer stem cells. Real-time PCR on sarcospheres revealed an increased expression of both *NANOG* and *OCT-4* in WF pool-stimulated cells compared to LS-treated cells (**P<0.01) (Supplementary Figure 3).

### Wound fluid promotes mesenchymal to endothelial transition

**Figure 4:**
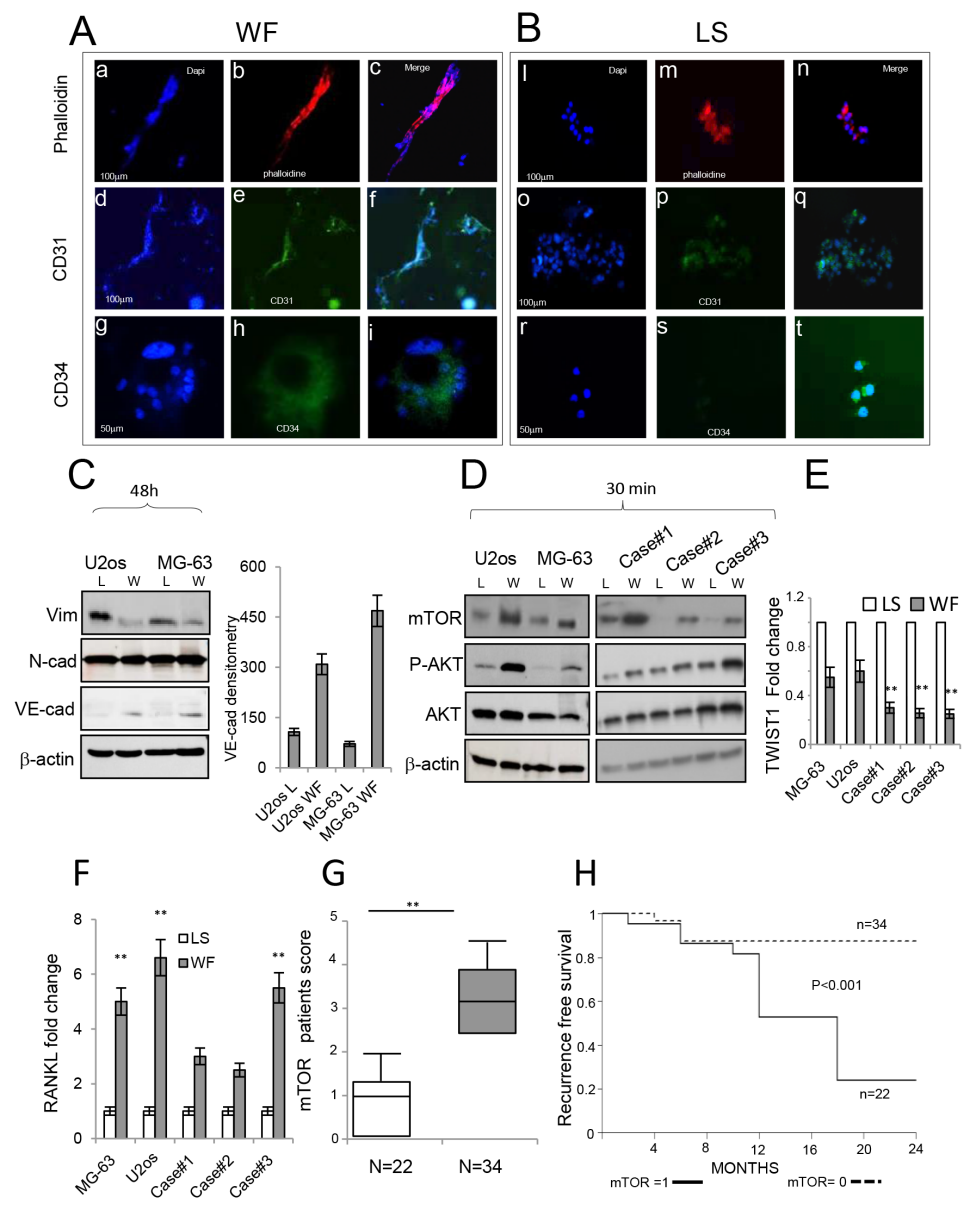
Role of WFs in the endothelial commitment of sarcoma cells **(A)** Immunofluorescence staining of MG-63 cells following WF pool stimulation at day 10. In panel (a) cells stained with DAPI, in panel (b) cells stained with phalloidin, in panel (c) merge image. Scale bar, 100 μm. In panel (d) cells stained with DAPI, in panel (e) cells stained with CD31 antibody, in panel (f) merge image. Scale bar, 100 μm. In panel (g) cells stained with DAPI, in panel (h) cells stained with CD34 antibody, in panel (i) merge images. Scale bar, 50 μm. **(B)** Immunofluorescence staining of MG-63 cells following LS stimulation at day 10 showing typical cell morphology. In panel (a) cells stained with DAPI, in panel (b) cells stained with phalloidin, in panel (c) merge images. Scale bar, 100 μm. In panel (d) cells stained with DAPI, in panel (e) cells stained with CD31 antibody, in panel (f) merge images. Scale bar, 100 μm. In panel (g) cells stained with DAPI, in panel (h) cells stained with CD34 antibody, in panel (i) merge images. Scale bar, 50 μm. **(C)** Representative Western blots (3 independent experiments) of total protein extracts from U2os and MG-63 cells following 48h of stimulation with LS or WF pool analyzed with antibodies as indicated. Actin was used as control of equally loaded samples. On the right, densitometry of VE-Cadherin. **(D)** Representative Western blot analysis of total protein extracts from U2os and MG-63 cells (left panel) and from 3 representative patient cell lines, following 30 min stimulation with LS or WF pool analyzed with antibodies as indicated (right panel). **(E)** Dosage of *TWIST1* gene by real-time PCR in established cell lines and 3 independent primary GCTB cell lines after stimulation for 48h with LS (white box) or WF pool (gray box). **(F)** Dosage of *RANKL* gene by real-time PCR in established cell lines and 3 independent primary GCTB cell lines after stimulation for 48h with LS (white box) or WF pool (gray box). **(G)** Box plot score of mTOR activation in patients. Median of mTOR expression in MG-63 cells considered equal to 1 was used as cut-off. mTOR lower than cut-off value (white box), mTOR above cut-off value (gray box). Statistical analyses were performed using the Student’s t-test: **P <0.01 vs LS. **(H)** Kaplan-Meier curves representing the relapse-free survival of 56 GCTB patients with low and high levels of mTOR following WF pool stimulation.

To further assess the transition of tumor cells into tubule-like structures, established cells were grown in presence of WF pool for 10 days and then analyzed by immunofluorescence microscopy. Since we observed similar results using MG-63 and U2os cell lines, here we report data only from MG-63 cells. Images of MG-63 cells grown in WF pool show that nuclei stained with DAPI were aligned (Figure 4A panel a), and cytoskeletons stained by phalloidin were fused, assuming tubule-like structures (Figure 4A panel b). These cells were focally positive to endothelial CD31 marker (Figure 4A panel d-f). Additionally, cells also formed circular structures, called rosettes, previously reported to form vessel lacunae *in vivo*, which showed a diffuse cytosolic positive staining to CD34 antibody (Figure 4A panels g-i). Cells grown in LS did not change their morphology, were only very slightly positive to CD31 and CD34, and tubule structures were not evident (Figure 4B panels i-t). In order to further understand the molecular mediators of wound fluids stimuli, we analyzed PI3K/AKT/mTOR pathway, previously reported involved in bone tissue renewal and stem cell differentiation into endothelial cells [22–25]. Western blot analysis indicated that at an early time point WF pool activated p-AKT and mTOR proteins, and after 48h reduced expression of mesenchymal markers such as Vimentin, and increased the endothelial marker VE-Cadherin (Figure 4D). Additionally, real-time PCR confirmed the reduction of another mesenchymal marker, *TWIST1* (Figure 4E), in cells stimulated with WF pool, and an increase in RANK ligand involved in osteoclast differentiation and GCTB pathogenesis (Figure 4F). More importantly, the reduction of mesenchymal proteins were also maintained after two weeks from stimulation, suggesting a stable modification. Since the mTOR protein was activated more strongly than p-AKT in patient cell lines, we evaluated its clinical significance. Primary GCTB cell lines from patients following WF pool stimulation were scored for mTOR by immunofluorescence, and the median of mTOR activation in MG-63 cells was used as cut-off value (Figure 4G). Kaplan-Meier analysis indicated that patients with mTOR above cut-off have high probability to develop recurrence (P< 0.001) (Figure 4H).

### Wound fluid induces tumor growth *in vivo*

**Figure 5:**
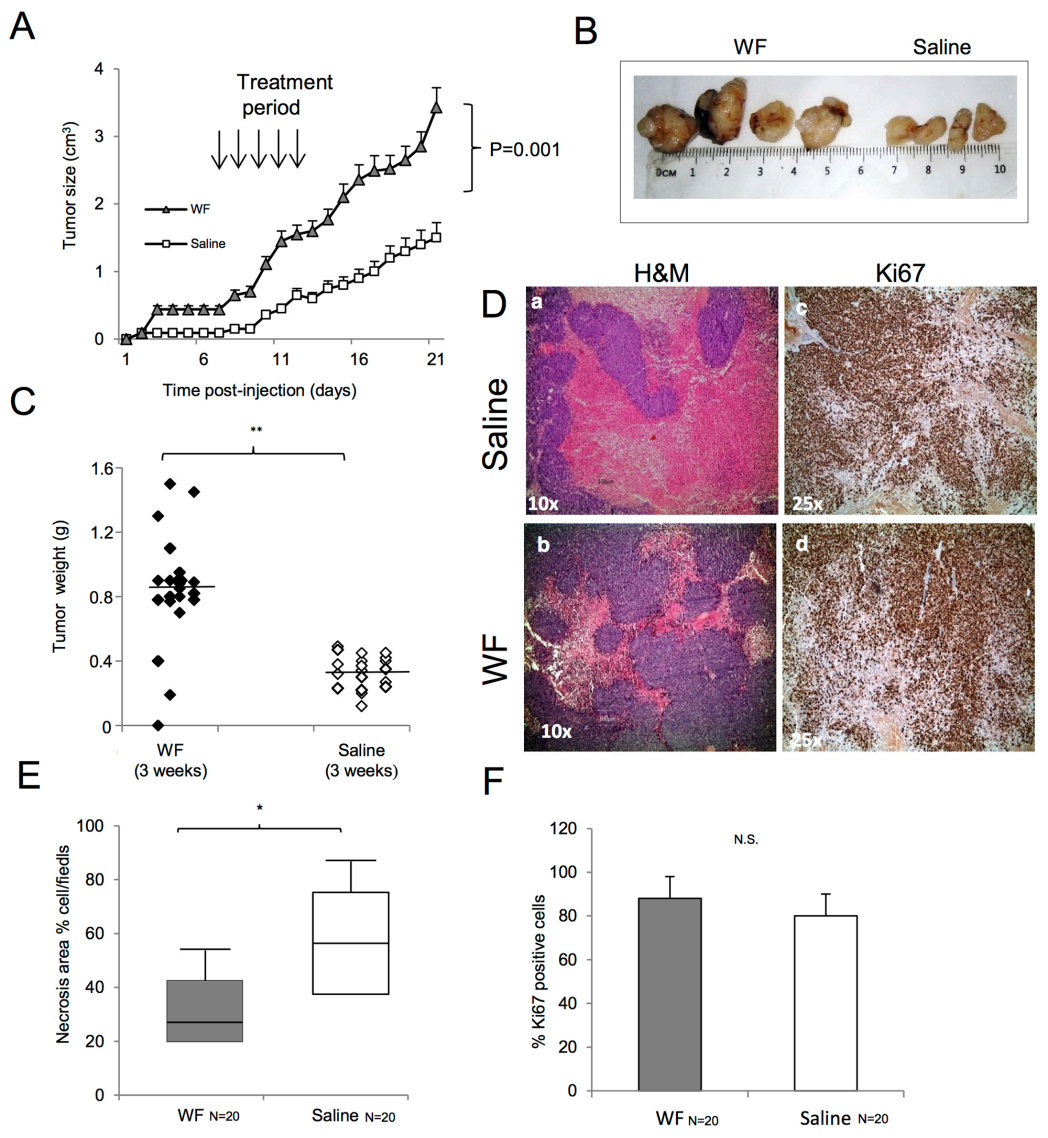
Malignant WF increases tumor growth in *in vivo* models **(A)** Tumor volume of subcutaneous mouse xenografts. One group of 10 mice was injected in both flanks with MG-63 cells and treated for 5 days with WF pool while control group (n=10) received saline solution. At day 21, differences between groups were determined by Mann-Whitney test; P <0.001. **(B)** Representative image of tumors from WF pool mice and saline-treated mice. **(C)** Tumor weight of subcutaneous mouse xenografts. Tumors developed in WF pool-treated mice (n=20, dark box), saline-treated group (n=20, white box). Differences between groups were determined by Mann-Whitney test: **P <0.01. **(D)** H&E staining of mice tumors: (panel a) treated with saline, (panel b) treated with WF pool at 10x magnification. Ki67 staining of mice tumors treated with saline (panel c), or with WF pool (panel d) at 25x magnification. **(E)** Box plot reporting percentage of necrotic cells; WF pool tumors (n=20, gray box) and saline group tumors (n=20, white box). Data report the mean value of H&M staining per field in 4 fields scored per section (n=20) by ImageJ program. Statistical analysis was performed with Student’s t-test: **P <0.01. **(F)** Bar graph reporting percentage of Ki67-positive cells per field in mice tumors: WF pool tumors (gray box), saline tumors (white box). Data report the mean value of 4 fields (n=20) scored by ImageJ program. Statistical analysis was performed with Student’s t-test: **P<0.01.

**Figure 6:**
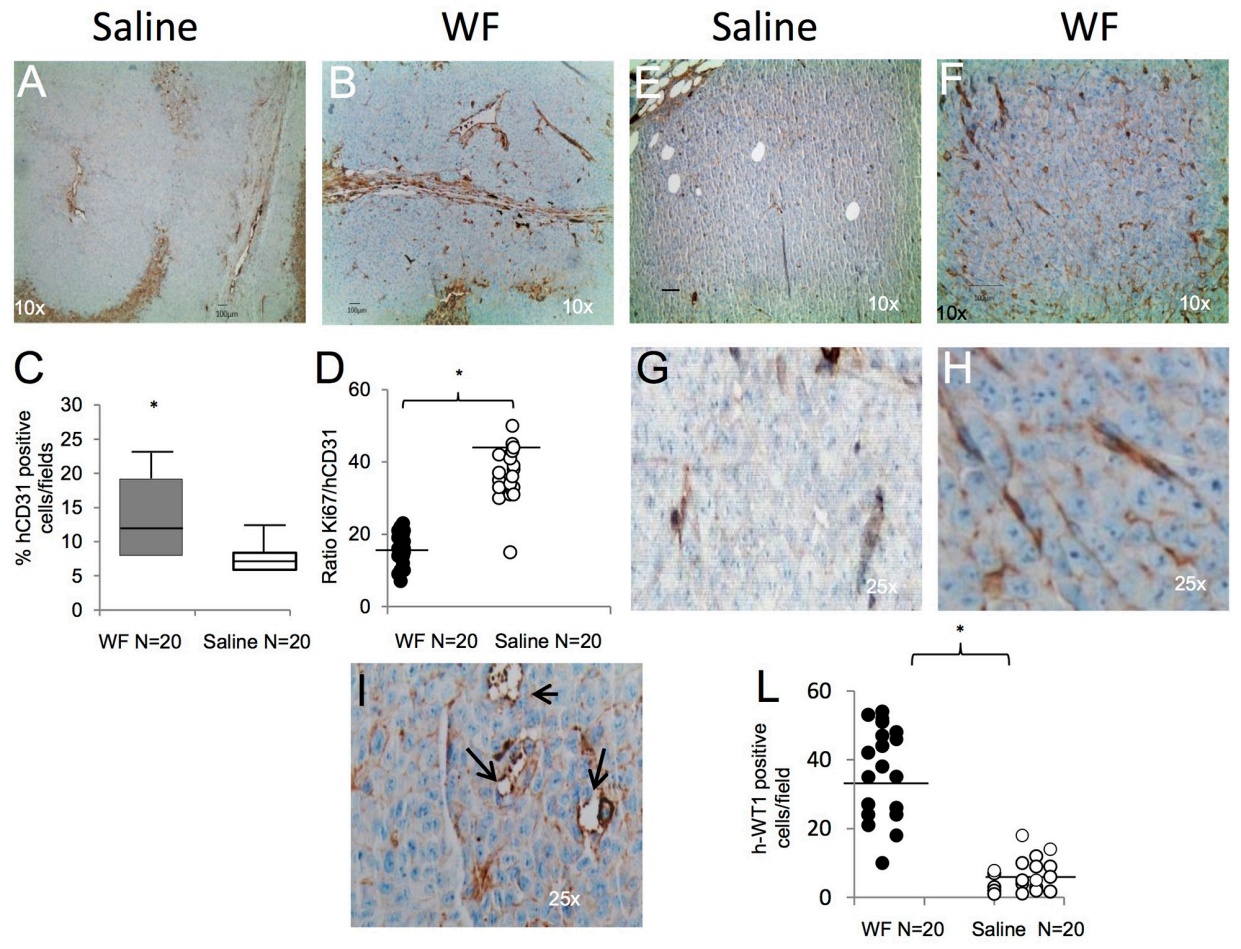
Tumor vessel determination and characterization **(A)** Representative image of human CD31 staining of tumor section from saline-treated mice at 10x magnification. **(B)** Tumor section from WF pool-treated mice stained with CD31 antibody at 10x magnification. **(C)** Box plot reporting hCD31-positive cells per field in WF pool tumors (n=20, gray box) and saline tumors (n=20, white box). Data report the mean value of 4 fields per section (n=20) scored by ImageJ program. Statistical analysis was performed with Student’s t-test: *P <0.05 vs saline treated mice. **(D)** Graph reporting ratio between Ki67 and hCD31-positive staining per field in WF pool (n=20, black circle) and in saline-treated tumors (n=20, white circle). Data report the mean value of 4 fields scored per section (n=20) by ImageJ program. Statistical analysis was performed with Student’s t-test: *P <0.05 vs saline-treated mice. **(E)** Representative staining with human WT1 antibody that detects neovessels in saline tumors, at 10x magnification. **(F)** Representative staining with human WT1 antibody in WF pool tumors, at 10x magnification. **(G)** Inset at 25x magnification of panel E. **(H)** Inset at 25x magnification of image in panel F. **(I)** Representative image of vessel lacunae present in WF pool-treated tumors. Arrows indicate lacunae at 25x magnification. **(L)** Graph reporting hWT1-positive cells per field in WF pool tumors (n=20, black circle) and in saline tumors (n=20, white circle): *P <0.05 vs saline treated mice. All data report the mean value of 4 fields scored per section (n=20) with ImageJ program and analyzed by Student’s t-test.

To assess the effect of WF pool on tumor growth *in vivo*, we injected human sarcoma cells in athymic nude mice and periodically measured tumor volume. Tumors injected/bathed with WF pool grew significantly faster than in saline-injected controls (P< 0.001). Additionally, tumor size between groups was significantly different during the period of fluid injections, with the largest difference of 2.65cm^3^ between WF pool group versus saline group at day 21 (P=0.034) (Figure 5A). Upon cessation of WF pool injections, tumor growth of the two groups remained different. Twenty days after tumor cell implantation, mice were euthanized and tumor growth was evaluated by tissue examination, tumor weight, and histopathology. A representative image of resected tumors highlights that mice group receiving WF pool develop tumors (n=20) larger than control group (Figure 5B) and with 2-fold higher weight (n=20;*P <0.05) (Figure 5C). To further investigate the role of WF pool in tumor recurrence, tumor tissues were analyzed by IHC. Hematoxylin and eosin (H&E) staining indicated that tumors treated with WF pool had lower necrosis areas (20%) per field compared to control group (40%) (Figure 5D panel a, b and 5E) while both tumors were strongly positive to Ki67 antibody (excluding necrosis area), indicating a high proliferation rate (Figure 5D panels c, d, and Figure 5F). To investigate whether WF pool affects tumor vascularization and in particular whether sarcoma cells trandifferentiate into endothelial cells *in vivo*, we measured the relative number of vessels of human origin with human CD31 antibody (Figure 6A and 6B). Tumors from mice group receiving WF pool had more hCD31-positive stained vessels compared to control (Figure 6A and 6B), in line with tumor mass/vessel density ratio (Figure 6C and 6D). The largest difference in tumor vascularization was observed with hWT1 antibody, able to detect early and immature vessels (neoangiogenesis). hWT1 antibody stained sections from WF pool tumors at 5-fold higher intensity than control (Figure 6E, 6F, and 6L), and depicted vessel lacunae as shown in Figure 6I. The presence of human- and mouse-derived endothelial cells was confirmed by flow cytometry analysis of xenographs, which counted an average of 5% cells positive to human CD31 and 2% to CD105 antibodies, and only 1% positive to mouse CD31 antibody (Supplementary Figure 4).

## DISCUSSION

Surgery is the gold standard procedure for GCTB, and leaves an acute wound to which the residual tumor cells are exposed [26]. Although, high recurrence rate of GCTB was correlated with surgical treatment [3, 27], the impact of post-surgery fluids on pathobiology of GCTB recurrence has not yet been fully investigated. The present work provides proof-of-concept data on the pro-tumoral role of post-surgery fluids from GCTB in primary GCTB stromal cells, established sarcoma cell lines and mouse model in influencing stem cell growth and AKT/mTOR pathway activation. Additionally, this study includes the screening of a cohort of human GCTB samples and evaluates mTOR activation as predictive of recurrence-free survival. We first isolated and characterized primary stromal cells from GCTB patients according to the standard procedure [20, 21]. In agreement with previous reports, our data confirmed that primary stromal GCTB cells are heterogeneous with mesenchymal lineage and display a pre-neoplastic phenotype [7, 12]. Our results clearly show that post-surgery fluids influence different aspects of primary GCTB and establish tumor cells plasticity. Specifically, post-surgery fluids increased the capability of cells to proliferate and migrate. In addition, WF pool enriched the expression of CD117^+^/CD44^++^ antigens, which become 30% and 75% of cell population, respectively. A high level of CD44^++^ antigen was previously described expressed in MSCs [28] and was associated with degree of malignancy of GCTB [29]. Similar results were reported for antigen CD117^+^, although detected at very low level (1% of cells) in human biopsies [12]. Our results indicate for the first time that post-surgery fluids affect CD44^++^ and CD117^+^ expression, increasing clonogenic and stemness property of cells assayed by sarcosphere formation and expression of *OCT-4* and *NANOG* genes.

In recent years, the origin of tumor vascularization was increasingly investigated [30, 31]. Reports document the heterogeneity of human endothelial tumor cells with markers common to pericytes [32, 33], bone marrow [34, 35], and tumor cells [36, 37], suggesting that tumor microenvironment induces their differentiation and enhances neovascularization [38]. However, the mechanisms that initiate phenotypic changes and drive mesenchymal-endothelial transition is yet to be defined [39, 40]. Our results highlight the possibility that post-surgery fluids contribute to neovascularization, promoting growth and transdifferentiation of cancer stem cells (CD44^++^/CD117^+^). Thus, upon prolonged exposure to post-surgery fluids, cells changed their morphology assuming a tubule-like phenotype positive to CD31 endothelial antigen, and increased VE-Cadherin. In accordance, post-surgery fluids reduced the mesenchymal markers *TWIST1* and Vimentin.

In order to better understand the mechanisms underlying the observed effects and to identify early molecular signal transduction pathways involved in this process, we stimulated at early time points both primary GCTB and established cells. Among the pathways strongly activated by post-surgery fluids we selected AKT/mTOR. AKT/mTOR is one of the most critical pathways in cancer stem cell growth [23, 24], and is implicated in transition of cancer stem cells to endothelial cells [25, 39, 40]. Thus, our results are in line with these reports and support AKT/mTOR activation as an early mediator of post-surgery tumor cell growth and plasticity. Interestingly, in a platform screening, mTOR was demonstrated overexpressed in over 2000 sarcomas [41]. Although, mTOR inhibitors were shown to be effective in various sarcoma cell lines [42], their efficacy in *in vivo* models is controversial [43, 44], and they displayed low benefit in clinical trials due to high toxicity [45, 46]. Of note, a new generation of mTOR-targeting agents were found to be effective in a preclinical model of leiomyosarcoma [47]. Our findings point to the clinical importance of AKT/mTOR pathway in GCTB patients as its overexpression was statistically correlated with higher recurrence rate by Kaplan-Meier logistic regression, albeit in a small cohort.

The functional significance of post-surgery fluids was further assessed *in vivo* using a mouse model. Tumors grew more rapidly in wound fluids-stimulated than in saline-stimulated mice, proving the concept that post-surgery fluids are a biologically potent component of tumor environment and are sufficient to promote tumor growth and neovascularization. Furthermore, since tumor size was larger among the post-surgery fluids-treated mouse group than the saline group, even after removal of fluids, it can be hypothesized that stable cellular modifications may occur. Interestingly, tumors from mice receiving post-surgery fluids presented a higher density of neovessels and vascular lacunae of human origin, and consequently lower necrosis areas than controls, pointing to transdifferentiation of human tumor cells into neovessels *in vivo*, as documented by human CD31^+^ cells in mice tumor. Taken together, these findings indicate that biological factors present in post surgery fluids are involved in: i) formation of the pre-recurrence niche promoting growth of CD117^+^ and CD44^++^ clonal stem cell populations; ii) transdifferentiation of MSCs into an endothelial-like phenotype, contributing to tumor neovascularization. These results provide further insights into the mechanism of recurrence, indicating that post-surgery fluids may be part of the relapse process. This novel observation supports further investigation into the use of mTOR inhibitors to explore their efficacy in preventing regrowth of GCTB. Our findings may also have new therapeutic implications since targeting the process of mesenchymal to endothelial differentiation could represent a treatment option before recurrence occurs.

## MATERIALS AND METHODS

### Primary cell culture and culture propagation

Established MG-63, U2os and Ost cell lines were obtained from American Type Culture Collection (ATCC) and cultured in Dulbecco’s Modified Eagle’s Medium (DMEM; Sigma) supplemented with 10% fetal bovine serum (FBS; Sigma), L-glutamine (4 mmol/L), 100 U/mL penicillin and 100 μg/mL streptomycin (all from Invitrogen) at 37°C with 5% CO2. For primary sarcoma cells, specimens were obtained in accordance with Research Ethics Board approval from the Italian National Institute of Cancer G. Pascale and classified according to World Health Organization guidelines. Primary GCTB cell line derived from patients were freshly isolated as previously described [19, 20] and grown in alpha-MEM (Sigma) supplemented with 10% FBS plus antibiotics. The experiments were performed in alpha-MEM plus 1% WF or 2% FBS (low serum) or 1% Benign effusion.

### Patients and wound fluid collection

A total of 56 biopsies and matched drainage fluids were collected from patients affected by GCTB (from 2010-2016 at the National Institute of Cancer G. Pascale. None of the patients recruited had other comorbidities or had received any radiotherapy or chemotherapy before the surgery. Clinical pathological characteristics and disease stage are indicated in Table 1. 50 mL drainage fluids (WFs) and benign fluids were collected in a sterile container without additives from GCTB and non-tumor patients, respectively, within 24h after surgery. WFs were centrifuged at 1600 RCF for 10 min, and then the supernatant was passed through a 0.22-μm filter and stored at −80°C. Written informed consent was obtained from individual patients, and the experimental protocol was approved by the local Ethics Committee. The assays were then performed using a single fluid or a pool of group fluids.

### Proliferation

For growth curve, 5x10^4^ cells/well were seeded in 96-well plates in alpha-MEM plus 2% FBS or 1% WF in triplicate. Our pilot experiments revealed 1% as the optimal concentration to assay WF biological effects. At the indicated times, MTT assay was performed following manufacturer’s instructions (Invitrogen). Absorbance was measured at 570 nm using a microplate reader (Titertek Multiskan PLUS, MK II, Labsystems).

### Colony and sarcosphere formation assay

Cells were plated at a density of 50, 200, and 500 cells per 60 mm dishes with alpha-MEM containing 2% FBS (low serum) or supplemented with WF pool (1%) for 2 weeks. The cells were stained with crystal violet dissolved with 10% glacial acetic acid and colonies >20 μm were assessed using a microplate reader (Titertek Multiskan PLUS, MK II, Labsystems,) set at 595 nm. Sarcosphere formation assay cells were plated at a density of 3000 cells/well in ultra-low attachment six-well plates in serum-free DMEM/F12 medium with N2 supplement (Gibco). Additional LS or 1% WF pool was added to the media every other day. After culture for 7–10 days, colonies were quantitated by inverted phase contrast microscopy. Five fields were counted for each well, and mean number and diameter of sarcospheres/field and calculated. A single-cell suspension derived from primary spheres was obtained at a plated density of 3000 cells/well for the secondary sphere formation using the same methods.

### Immunofluorescence staining and immunohistochemistry

Cells were plated onto glass coverslips and fixed with 4% paraformaldehyde in PBS for 15 min, and permeabilized for 10 min with 0.2% Triton in PBS containing normal goat serum (blocking solution). Primary MAb antibody incubation was performed for 1h at room temperature, and antibodies used were: CD31 (ab28364; Abcam), CD34 (EP373Y; ab81289; Abcam) or Alexa Fluor 647 Phalloidin (A22287; Life Technologies). Secondary antibodies, incubated for 45 min, were FITC-Alexa Fluor 488 (A21206; Life Technologies). Cell nuclei were counter-stained with DAPI (Sigma). Images were evaluated by Leica microscope and photographed using a Nikon DXM1200 digital camera. The resulting images were white-balanced using Photoshop CSsoftware; no further image manipulation was performed. Tumor sections of 4-5μm of tumors were deparaffinized and stained with H&E and then with CD31 (JC/70; Dako Corporation) and WT1 (6F/H2; Ventana Medical Systems Inc.) and Ki67 (Dako Corporation). All the antibodies were used at 1:100 dilution. The sections were then stained using avidin-biotin complex by immunoperoxidase technique. Patients were scored for mTOR activation using as cut-off value the median of mTOR activation in MG-63 cells cultured with WF pool =1. Labeling index and total number of nuclei (blue) in each field were scored and the ratio of two values was automatically calculated by Image-Pro Plus 6.0 software under Leica microscope.

### Western blot

Proteins were extracted and separated by 15% SDS–PAGE gel using the standard technique [21]. Membranes were incubated with the following antibodies: Vimentin (V9; Abcam), Phospho-AKT (Ser473, D9E; Cell Signaling Technology), VE-Cadherin (ab33168 abcam), m-TOR (sc-293089) and N-cadherin (H63) (all from Santa Cruz Biotechnology), and β-actin (Cell Signaling Technology). Image analysis was performed by ImageJ software (http://rsbweb.nih.gov/ij/).

### Cell sorting analysis

For cell sorting analysis, 1x10^5^ cells were sorted with the following antibodies: anti-CD44-FITC (R&D Systems), H-CAM, (clone IgG2b IM7), FITC anti-human CD45 (clone IgG1 HI30), FITC anti-human CD73 (clone AD2), Per-CP anti-human CD90 (clone Thy-1, clone 5E10), PE anti-human CD105 (clone IgG1 SN6; all from Biosciences), human CD117-PE (clone REA787), human CD31-FITC (clone REA730), human CD34-PE (clone AC136; all from Miltenyi Biotec), PE/Cy7 anti-mouse CD105 (clone MJ7/18) and PE anti-mouse CD31 (clone 390, both from BioLegend). Twenty thousand event cells were analyzed at each experimental point using FACS ARIA II (Becton Dickinson). Background fluorescence was estimated by substituting primary antibody with specific isotype controls.

### Semiquantitative and quantitative real-time PCR

To assess mRNA expression levels of embryonicstem cell markers, total RNA was extracted using Trizolreagent (Invitrogen) followed by RNeasy Mini Kit(Qiagen Inc.). Reverse transcription was performed usinga SuperScript First-Strand Synthesis Kit (Invitrogen). For semiquantitative PCR, 1 μL of target cDNA conversion mixturewas amplified using Hotstar Taq DNA Polymerase (Qiagen) for 35cycles at 94°C for 30 seconds, at 55°C for 30 seconds, and at 72°Cfor 1 min. For primers, see Supplementary Table 2.

### *In vivo* animal model

Human sarcoma MG-63 cells (10^6^/mouse) were injected subcutaneously into two flanks of 6–8-week-old NOD/SCID nude mice (Charles River). After 5 days, mice were divided into two groups of 10. One group received loco-regional subcutaneous injections of WF pool (100 μL/daily for 5 days). The other group received phosphate buffered saline allowed by local Ethics Committee guidelines. Tumor size was determined by microcallipers and calculated according to the formula: tumor volume (mm^3^) = L×W2/2, where L is the length and W is the width. After 21 days, the mice were sacrificed by CO2 asphyxiation. Tumors were excised immediately and weighed or stored in 10% paraffin for following IHC. The animal experiments were approved by the Animal Ethics Committee of the Italian Ministry of Health (764/2016-PR).

### Statistical analysis

Statistical analysis was performed using GraphPad (GraphPad Software Inc.) and R software version 3.1.0 (R Foundation for Statistical Computing). Comparisons of continuous variables between the two groups were performed by unpaired Student’s t-test or Mann-Whitney test according to their distribution. Tumor growth curves were analyzed by means of repeated measurements by ANOVA with Bonferroni post-hoc test. Patients were scored for mTOR activation using as cut-off value the median of mTOR activation in MG-63 cells cultured with WF pool. Patient cell lines with mTOR above the median, following WF pool exposure, were scored =1 (n=22). Progression-free survival curves were estimated by Kaplan–Meier method and compared with log-rank test. A two-tailed P value <0.05 was considered significant.

## SUPPLEMENTARY MATERIALS FIGURES AND TABLES


