# Correction: Postsurgery fluids promote transition of cancer stem cell toendothelial and AKT/mTOR activity contributing to relapse of giant cell tumors of bone

**DOI:** 10.18632/oncotarget.28872

**Published:** 2026-05-04

**Authors:** Flavio Fazioli, Gianluca Colella, Roberta Miceli, Mariano Giuseppe Di Salvatore, Michele Gallo, Serena Boccella, Annarosaria De Chiara, Carlo Ruosi, Filomena de Nigris

**Affiliations:** ^1^Division of Musculoskeletal Oncology Surgery, National Cancer Institute G. Pascale, Naples, Italy; ^2^Department of Human Health, Federico II University of Naples, Naples, Italy; ^3^S.C. Cellular Biology and Biotherapy, National Cancer Institute G. Pascale, Naples, Italy; ^4^Department of Experimental Medicine, University of Campania “Luigi Vanvitelli”, Naples Italy; ^5^Division of Pathology, National Cancer Institute G. Pascale Foundation, Naples, Italy; ^6^Department of Biochemistry, Biophysics and General Pathology, University of Campania “Luigi Vanvitelli”, Naples Italy; ^*^These authors have contributed equally to this work

**This article has been corrected:** It was found that Figure 3C (upper panel) was identical to Figure 5E in a 2013 article published by the same group [1]. The authors explained that an image of endothelial cells was incorrectly uploaded instead of the intended image showing patient tumor cells after three days of stimulation. Due to the similarity of the structures formed in these different experiments, the error was previously overlooked.

The authors have provided the correct image, including the original metadata and snap date, to confirm its authenticity. The original description in the paper remains accurate, and the interpretation of the results is unaffected by this correction.

The correct Figure 3 is presented below.

Original article: Oncotarget. 2017; 8:85040–85053. 85040-85053. https://doi.org/10.18632/oncotarget.18783

**Figure 3:**
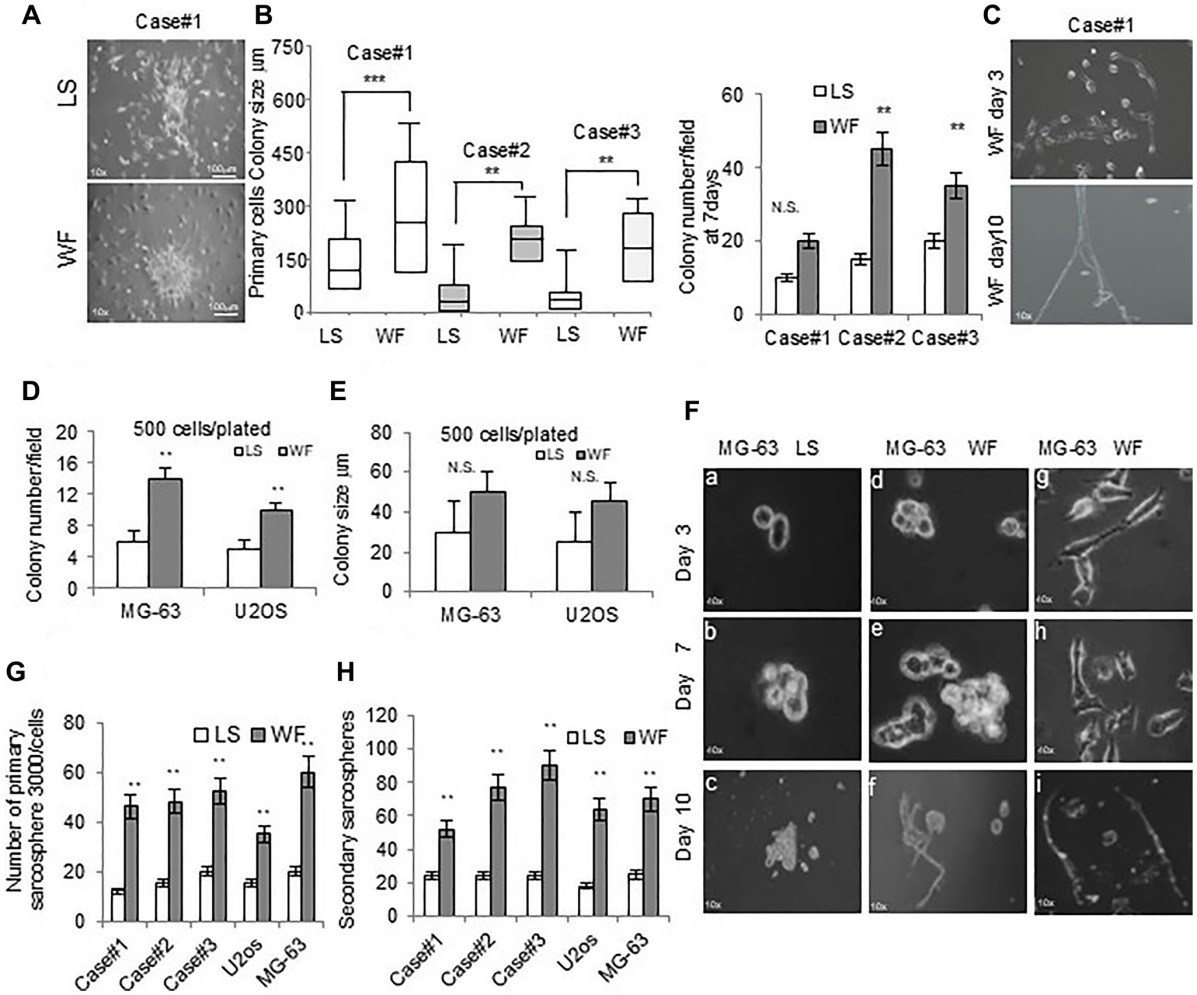
Effect of WF pool colony formation and sarcospheres. (**A**) Representative colony clusters formed by primary GCBT cell line stimulated for 48h with LS (upper panel) and with WF pool (lower panel) at 10x magnification. (**B**) Left panel, box plots reporting colony size per field (500 cells plated) of 3 different primary GCTB cell lines in LS and WF pool as indicated after 48 h. right panel, colony number per field of 3 different primary GCTB cell lines in LS and WF pool as indicated after 7 days. Mean ± SD of 3 independent experiments: ^**^P < 0.01; ^***^P < 0.001. (**C**) Image of primary GCTB cell line grown in WF pool showing a subpopulation of cells with a cobblestone morphology at day 3 (upper panel) and after 10 days (lower panel) at 10x magnification. (**D**) Bar graph of MG-63 and U2os colony numbers per field, 500 cells plated, grown for 7 days in presence of LS (white box) or WF pool (gray box). The cells were stained with crystal violet and colonies >20 μm were counted. (**E**) Bar graph reporting colony size per field (500 plated cells) of MG-63 and U2os cells grown for 7 days in presence of LS (white box) or WF pool (gray box). (**F**) Sarcosphere assay of MG-63 cells grown in LS (panels a–c) and WF pool at different time points as indicated (panels d–i) at 10x magnification. At day 7 colonies were evident (panels b, e), and in presence of WF pool spontaneously differentiated into a tubular-like phenotype at day 10 (panels f) at 10x magnification. Panels (g–i) a subpopulation of MG-63 cells grown in WF pool showing cobblestone morphology and organized in tubular-like structures at day 10 (panel i). 10x magnification. (**G**) Number of spheres (>20 μm in diameter) generated after 7 days from 3000 plated cells treated with WF pool compared with LS group. (**H**) 3000 single cells isolated from primary spheres were cultured in sphere-forming specific system with WF pool or LS to form secondary spheres. The number of secondary spheres after 7 days (>20 μm in diameter) are reported. All the experiments were performed in triplicated and statistical analyses were performed using the Student’s *t*-test: ^**^P < 0.01; ^***^P < 0.001.
