# Supplementary material for: Post-surgery fluids promote transition of cancer stem cell-to-endothelial and AKT/mTOR activity, contributing to relapse of giant cell tumors of bone
Source: Oncotarget. 2017 Jun 28;8(49):85040–53. doi: 10.18632/oncotarget.18783 (PMC5689592; doi:10.18632/oncotarget.18783)
Supplement: Supplementary file 1 [file oncotarget-08-85040-s001.pdf]

Post-surgery fluids promote transition of cancer stem cell-to-endothelial and AKT/mTOR activity, contributing to relapse of Giant Cell Tumors of Bone

SUPPLEMENTARY MATERIALS

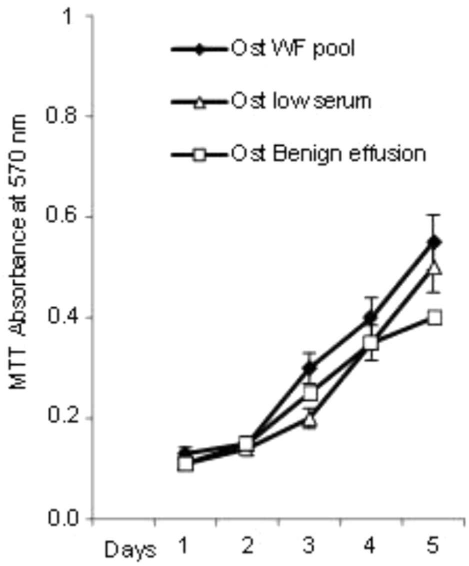

Supplementary Figure 1: Sarcoma wound fluids do not stimulate proliferation of osteoblastic cells. Ost cell line growth in WFs (black square) LS (white triangle) and Benign effusion (white square) dosed by MTT assay and reading the absorbance at 570 nm (mean ± SD of 3 independent experiments).

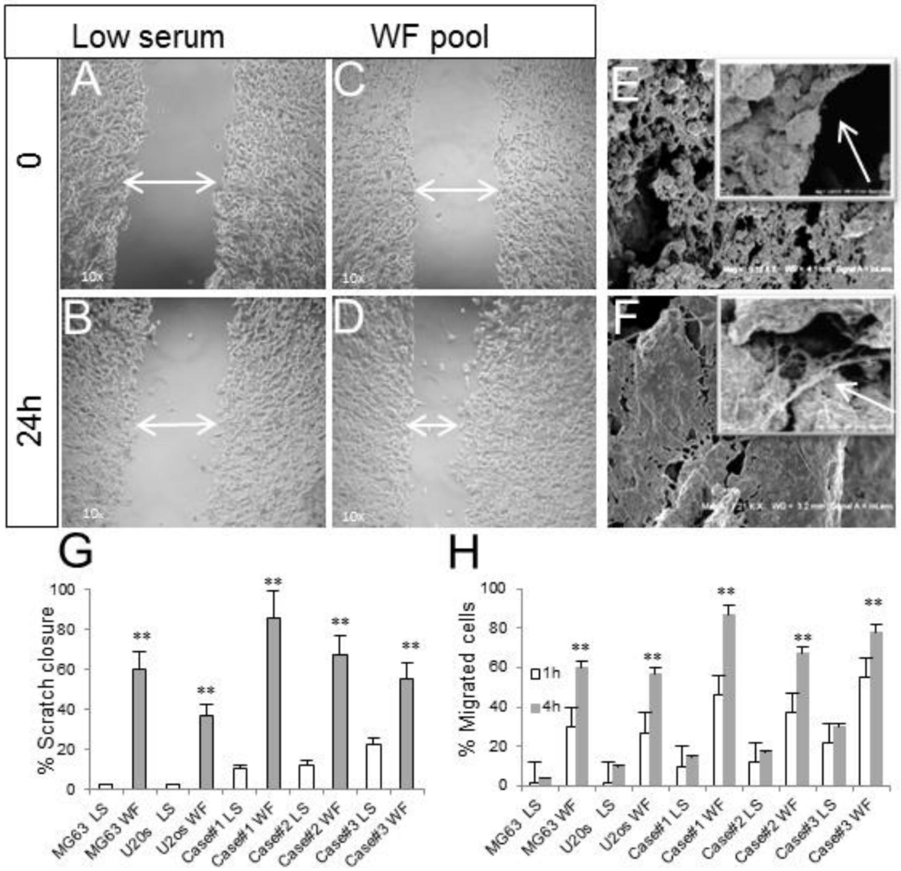

Supplementary Figure 2: Sarcoma wound fluids stimulate migration of sarcoma cells. **(A and B)** Representative images of MG-63 before and after 24h stimulation with Low serum (LS). **(C and D)**. Representative images of MG-63 before and after 24h stimulation with pool of wound fluids from primary tumor patients (WF; n=20). The images panel are captured manually with the Nikon Coolpix 4500, Nikon Corp. (Tokyo, Japan) at 10× Magnification. The rate of wound closure was determined by the Cellavista imager, **P< 0.001 (mean ± SD of 3 independent experiments). **(E)** SEM microscopy analysis of MG-63 cultured for 24h on silicate granules in presence of LS appeared to be well attached to the substrate with small pseudopodias (inset at scale bar: 10μm). **(F)** MG-63 cells after 48h with wound fluid pool. The arrows indicated long pseudopodias like structures (inset at scale bar: 10μm). **(G)** Bar graph reported the percentage of scratch closure after 24h determined by the CellAvista imager,**P< 0.001vs LS (mean ± SD of 3 independent experiments). **(H)** Bar graph of migration assay performed using the Transwell assay *in vitro*. MG-63, an U2os were placed on Transwell plates, in wound fluid pool from primary tumor patients (WF; n=20) or Low serum (LS; n=9) were added to the lower chamber of migration. After 24h the percentage of migrated cells in lower chamber were stained and counted. WF-treated cells migrated in a number significant higher than that in the control group.**P < 0.001 vs LS (mean ± SD of 3 independent experiments).

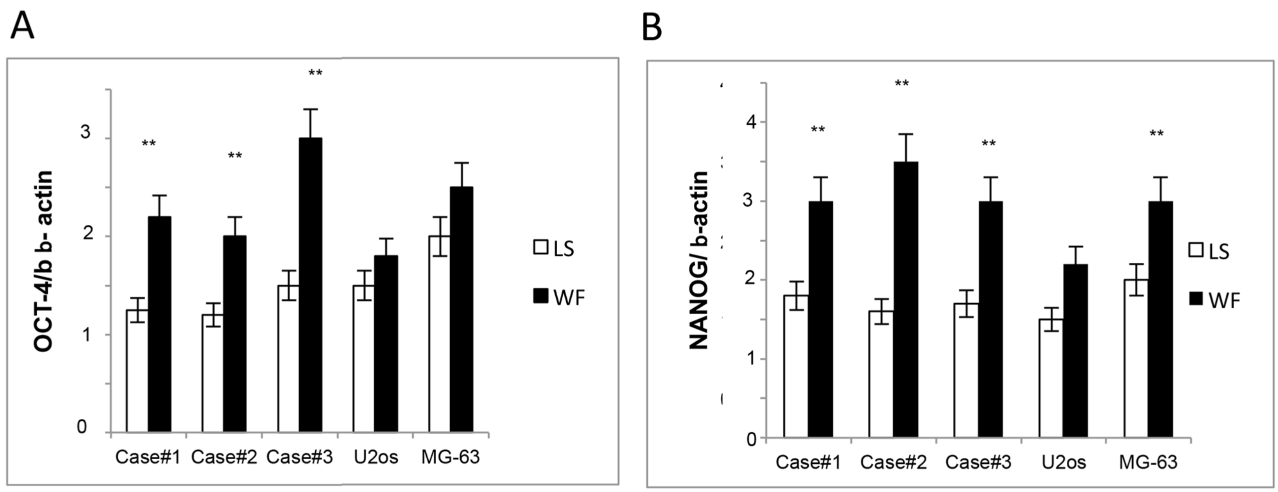

Supplementary Figure 3: Sarcoma wound fluids stimulate expression of stem cell markers. (A) Expression by real time PCR of OCT-4B gene and in **(B)** NANOG in spheres from primary GCTB cells, case#1, case#2, case#3, and U2os, MG-63 cells treated with WF pool and LS. **P<0.01vs LS (mean ± SD of 3 independent experiments).

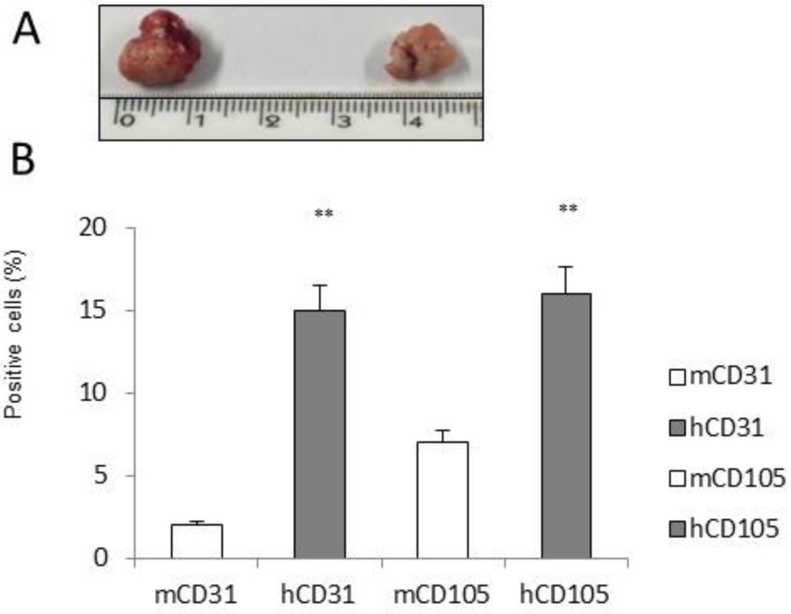

Supplementary Figure 4: Vessels of both human and mouse origin. (A) Representative image of mice saline tumors (right) and Wound fluid pool WF (left). **(B)** Bar graph reporting percentage of positive cells (extracted from tumors WF or saline treated) to mouse and human CD31 and CD105 antibodies analyzed by FACs; **P <0.01 (mean ± SD of 3 independent experiments).

Supplementary Table 1: Post surgery sarcoma fluid biochemical characteristics

Diagnosis	Glucose (nmol/L)	pH	LDH (U/L)	Protein (g/L)
GCTB primary tumor (n=38)	100.5 (0.1–10.4)	7.3 (7.0–7.5)	317.5(125.0-6497)	5.32 (10.0–58.0)
Recurrence (n=18)	105.5 (0.8–9.1)	7.4 (7.1–7.6)	438.0 (99.0–239.0)	4.0 (10.0–58.0)

Supplementary Table 2: Primers used in PCR assays

Gene	Accession number	Primer sequence
RANKL	NM.19147	Forward 5′-AAGGAGCTGTGCAAAAGGAATT-3′ ;
		Reverse 5′-TGATGTGCTGTGATCCAACGA-3′ ;
Twist 1	NM.000474.3	Forward: 5’-TCACGAGCGGCTCAGCTAC-3;’
		Reverse: 5’-TCTCTGGAAACAATGACATCTAGGTC-3;’
Oct3/4B	NM.203289	Forward: 5’-AGT CAG TGA ACA GGG AAT GG-3’;
		Reverse: 5’-TCG GGA TTC AAG AAC CTA CG-3’,
Nanog	BC_099704	Forward: 5’-TCC TCC TCT TCC TCT ATA CTA AC-3’;
		Reverse: 5’-CCC ACA AAT CAC AGG CAT AG-3’,
β-actin	BC_013380	Forward: 5’-GCG GGA AAT CGT GCG TGA CATT-3’;
		Reverse: 5’-GGC AGA TGG TCG TTT GGC TGA ATA-3’,
